# Targeted Metabolomic Analysis of a Mucopolysaccharidosis IIIB Mouse Model Reveals an Imbalance of Branched-Chain Amino Acid and Fatty Acid Metabolism

**DOI:** 10.3390/ijms21124211

**Published:** 2020-06-12

**Authors:** Valeria De Pasquale, Marianna Caterino, Michele Costanzo, Roberta Fedele, Margherita Ruoppolo, Luigi Michele Pavone

**Affiliations:** 1 Department of Molecular Medicine and Medical Biotechnology, School of Medicine, University of Naples Federico II, 80131 Naples, Italy; valeria.depasquale@unina.it (V.D.P.); marianna.caterino@unina.it (M.C.); michele.costanzo@unina.it (M.C.); luigimichele.pavone@unina.it (L.M.P.); 2 CEINGE—Biotecnologie Avanzate s.c.ar.l., 80145 Naples, Italy; fedeler@ceinge.unina.it

**Keywords:** lysosomal storage diseases, mucopolysaccharidosis IIIB, metabolomics, branched-chain amino acids, acylcarnitines, fatty acids

## Abstract

Mucopolysaccharidoses (MPSs) are inherited disorders of the glycosaminoglycan (GAG) metabolism. The defective digestion of GAGs within the intralysosomal compartment of affected patients leads to a broad spectrum of clinical manifestations ranging from cardiovascular disease to neurological impairment. The molecular mechanisms underlying the progression of the disease downstream of the genetic mutation of genes encoding for lysosomal enzymes still remain unclear. Here, we applied a targeted metabolomic approach to a mouse model of PS IIIB, using a platform dedicated to the diagnosis of inherited metabolic disorders, in order to identify amino acid and fatty acid metabolic pathway alterations or the manifestations of other metabolic phenotypes. Our analysis highlighted an increase in the levels of branched-chain amino acids (BCAAs: Val, Ile, and Leu), aromatic amino acids (Tyr and Phe), free carnitine, and acylcarnitines in the liver and heart tissues of MPS IIIB mice as compared to the wild type (WT). Moreover, Ala, Met, Glu, Gly, Arg, Orn, and Cit amino acids were also found upregulated in the liver of MPS IIIB mice. These findings show a specific impairment of the BCAA and fatty acid catabolism in the heart of MPS IIIB mice. In the liver of affected mice, the glucose-alanine cycle and urea cycle resulted in being altered alongside a deregulation of the BCAA metabolism. Thus, our data demonstrate that an accumulation of BCAAs occurs secondary to lysosomal GAG storage, in both the liver and the heart of MPS IIIB mice. Since BCAAs regulate the biogenesis of lysosomes and autophagy mechanisms through mTOR signaling, impacting on lipid metabolism, this condition might contribute to the progression of the MPS IIIB disease.

## 1. Introduction

Lysosomal storage diseases (LSDs) are a group of hereditary metabolic disorders which are inherited in an autosomal recessive manner, except for some that are X-linked [1]. They are caused by mutations in gene encoding for enzymes involved in the degradation of various macromolecules. The substrate(s) of the defective enzyme(s) building up over time leads to excess cellular storage of this material, which results in altered cellular functions, tissue damage, and organ dysfunctions [2]. As well as the accumulation of undigested or partially digested macromolecules in lysosomes, pathological features common to LSDs encompass the secondary storage of toxic metabolites, impairment of lipid trafficking, signaling dysregulation, enhanced inflammation, perturbed calcium homeostasis, endoplasmic reticulum stress, and activation of the unfolded protein response [3]. Altogether these dysfunctions culminate in altered autophagy, accelerated apoptosis, and cell death [4,5].

Among the LSDs, the mucopolysaccharidoses (MPSs) are eleven inherited metabolic diseases caused by a deficiency of the lysosomal enzymes needed to catabolize glycosaminoglycans (GAGs) [6]. A progressive multisystem disease characterizes each of these disorders, with key morbid manifestations involving the skeleton, joints, somatic tissues, heart, and, in some cases, the central nervous system (CNS) [6,7,8,9,10,11,12]. Although classical in its use, the nosology of the MPSs, based on the deficient enzyme and the accumulated GAGs, does not reflect the true nature of the complexity of these disorders. Animal models and studies in humans demonstrated that impaired GAG degradation causes a deep alteration of cellular homeostasis involving pathways unrelated to the GAG metabolism [10,11,13,14,15,16]. Indeed, defects in the lysosomal degradation of GAGs can be associated with dysfunctions in several biochemical and physiological processes such as the abnormal composition of membranes with an impact on vesicle fusion and trafficking, altered ganglioside and cholesterol metabolism, proteinase deregulation, impairment of autophagy and mitochondrial functions, and others [14,15,16,17,18,19,20]. Thus, a more detailed understanding of the pathogenetic events downstream of the altered GAG metabolism is still required.

The autosomal recessive disorder MPS IIIB caused by mutations in the gene encoding for the α-n-acetylglucosaminidase (NAGLU, enzyme entry: EC 3.2.1.50) enzyme involved in the degradation of heparan sulfate (HS) is one of the four MPS III (Sanfilippo syndrome) subtypes [6]. Patients affected by MPS IIIB exhibit profound mental retardation, behavioral problems, skeletal abnormalities, cardiovascular dysfunctions, and death usually in the second decade, along with somatic manifestations that are highly variable among the different phenotypes [12]. A murine model of MPS IIIB was generated through the targeted disruption of the *NAGLU* gene [21]. These mice exhibit a massive increase in HS deposition in the liver and kidney and, to a lesser extent, in the lung, spleen, thymus, and heart. This mouse model has been demonstrated to represent a useful tool for pathogenetic studies and therapeutic approach testing. We recently performed a quantitative proteomic analysis of the MPS IIIB mouse brain, which allowed the identification of the energetic metabolism among the perturbed processes in affected brains [22]. However, beside central nervous system disorders, cardiac and liver involvement in MPS IIIB has been demonstrated that contributes to the progression and poor outcome of the disease [6,9,23,24,25].

Metabolomics is an emerging “omics” science that represents an appealing and useful tool to determine changes in the profile of metabolites in living systems resulting from environmental conditions and/or genetic backgrounds [26,27,28]. To date, few studies have explored the metabolic phenotype in MPSs. Serum global metabolomic profiling revealed disturbances in the amino acid, peptide, carbohydrate, lipid, nucleotide, vitamin, cofactor, and xenobiotic metabolism in MPS IIIA and IIIB patients [29]. Furthermore, targeted and untargeted metabolomic analyses of urine samples from MPS I-, MPS III-, and MPS VI-affected patients showed impairment in the arginine, proline, histidine, and glutathione metabolism [30,31,32]. Here, we applied a targeted metabolomic approach to identify impaired amino acid and fatty acid metabolic pathways or other metabolic phenotypes in the liver and heart of the NAGLU^−/−^ mouse model of the MPS IIIB disease. The analysis of the metabolite profile and the identification of the main impaired metabolic processes affected by the absence of NAGLU in the heart and liver tissues of mice might provide further insights into the molecular mechanisms underlying the onset and progression of the disease, thus helping the search for an effective cure of such intractable disease.

## 2. Results

### 2.1. Metabolomic Profiles of Heart and Liver of MPS IIIB Mice

A targeted metabolic analysis was performed to evaluate changes in the concentration of Ala, Val, Xle (Leu and/or Ile), Met, Phe, Tyr, Asp, Glu, Gly, Orn, Cit, and Arg amino acids and saturated, unsaturated, hydroxylated and branched acylcarnitines in the heart and liver of NAGLU^−/−^ mice compared to age-matched wild-type (WT) mice.

**Figure 1:**
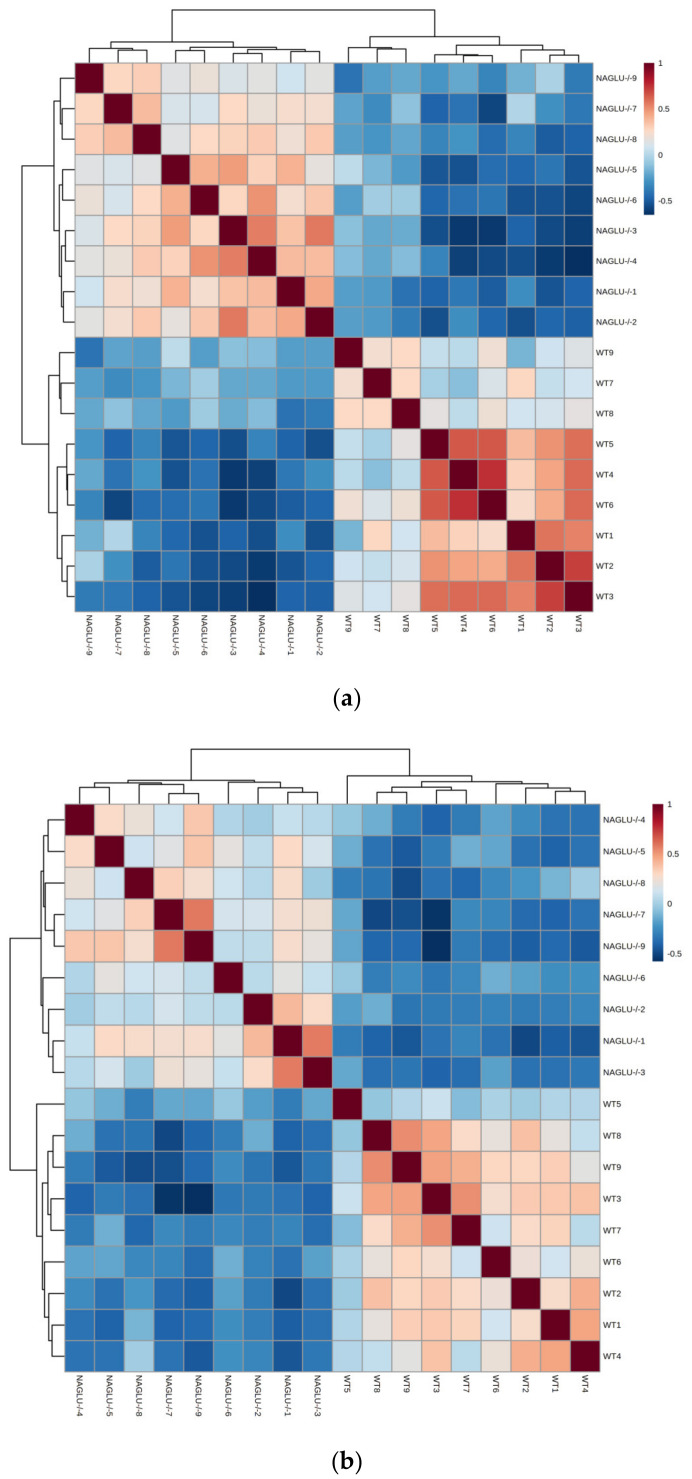
Correlogram of intra- and inter-groups (NAGLU^−/−^ and WT) from heart (**a**) and liver (**b**) tissues. The concentrations of the metabolites from NAGLU^−/−^ and WT heart and liver were normalized according to protein tissue contents, log(2) transformed, Pareto scaled, and analyzed using Spearman’s rank correlation analysis with MetaboAnalyst 4.0 software. The red and blue colors of the cells in the heatmaps indicate positive and negative correlation, respectively. Abbreviations: NAGLU, α-n-acetylglucosaminidase; WT, wild type.

Firstly, in order to test the power of the comparative studies performed, we carried out Spearman’s rank correlation analysis to evaluate sample reproducibility within the groups based on nine animals for each genotype for each tissue. As expected, the overall analysis displayed a positive intragroup correlation and a negative intergroup correlation in both the analyzed tissue-metabolomes (Figure 1a,b).

**Figure 2:**
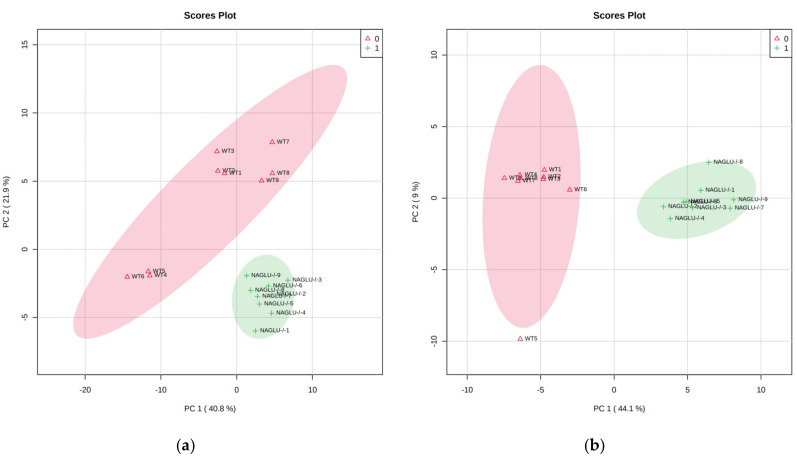
A principal component analysis (PCA) was performed using 48 metabolites from the heart (**a**) and liver (**b**) tissues. In NAGLU^−/−^ heart, the first two principal components (PCs) explained 62.7% of the total variance (PC1 40.8%, PC2 21.9%). In NAGLU^−/−^ liver, the first two PCs explained 53.1% of the total variance (PC1 44.1%, PC2 9.0%).

Moreover, the datasets, corresponding to the 48 analyzed metabolites (Tables S1 and S2), were processed by principal component analysis (PCA) to evaluate the difference rate and variance occurring between the genotype groups (NAGLU^−/−^ and WT) in the metabolic profiles characterizing the heart and liver. The PCA score plot revealed a clear separation between the analyzed data groups for each tissue (heart: principal component (PC) 1 variance 40.8%, PC2 variance 21.9%; liver: PC1 variance 44.0%, PC2 variance 9%). Outliers were not found in the heart and liver metabolomes (Figure 2a,b).

**Figure 3:**
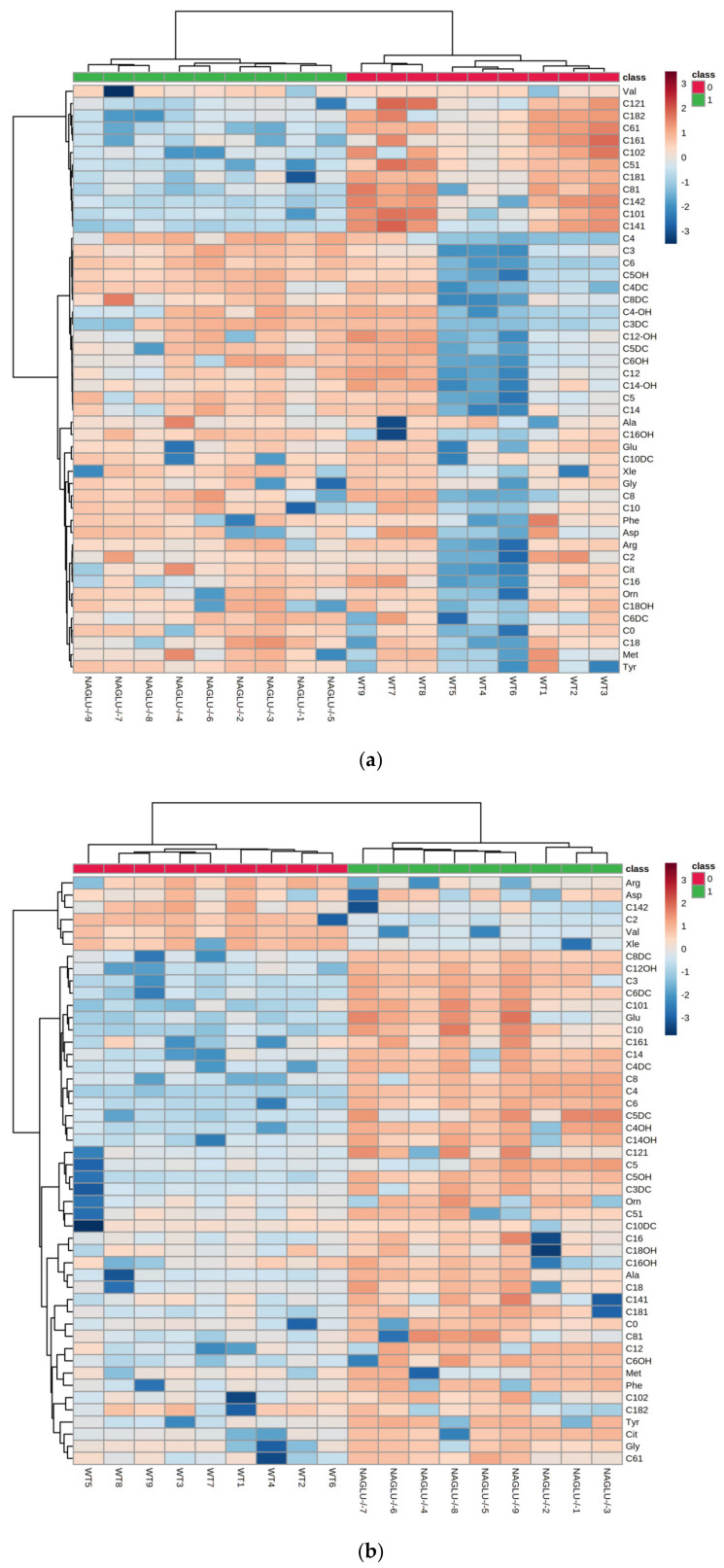
Hierarchical cluster analysis and heatmap of the metabolites in the (**a**) heart and (**b**) liver tissues from NAGLU^−/−^ and WT mice. The concentrations of the metabolites from NAGLU^−/−^ and WT heart and liver were normalized according to protein tissue contents, log(2) transformed, and Pareto scaled. The color code in the heatmap represents the relative metabolite abundance: red and blue colors mean increased and decreased levels of each metabolite in NAGLU^−/−^ versus WT, respectively.

To further understand the alterations in NAGLU knockout-related metabolites in the heart and liver, we used a heatmap to visually represent the differentially abundant metabolites in NAGLU^−/−^ and WT genotypes for each tissue; the concentrations of the metabolites were ranked by the t-test (*p* < 0.05 cut-off). The results showed correct sample group clusterization, and the dendrogram structure, using Euclidean distance, resulted in two main clusters, relating to the compared samples (NAGLU^−/−^ and WT) and metabolites, respectively (Figure 3a,b).

The heatmaps exhibit distinct patterns of metabolites between MPS IIIB mice and age-matched WT mice in both the analyzed tissues (heart and liver). Thus, the detected metabolic abnormalities are likely to be associated with the MPS IIIB phenotype, allowing the identification of a pathological metabolomic signature of the MPS IIIB disease.

### 2.2. Heart- and Liver-Specific Metabolomic Changes in MPS IIIB Mice

With a univariate analysis, we detected significant differences in bot amino acid and acylcarnitine levels in the liver and heart of MPS IIIB mice when compared to WT.

**Figure 4:**
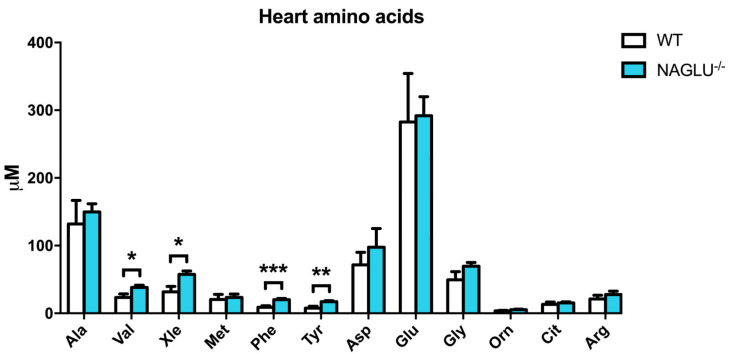
Branched-chain and aromatic amino acid accumulation in the heart of NAGLU^−/−^ mice. Results are represented as the mean ± standard error of the mean (SEM). The statistical significance of the difference in metabolite tissue concentrations between genotype conditions (NAGLU^−/−^ and WT) was evaluated by parametric or non-parametric tests when data failed the Shapiro–Wilk normality test. * *p* < 0.05, ** *p* < 0.01, *** *p* < 0.001.

**Figure 5:**
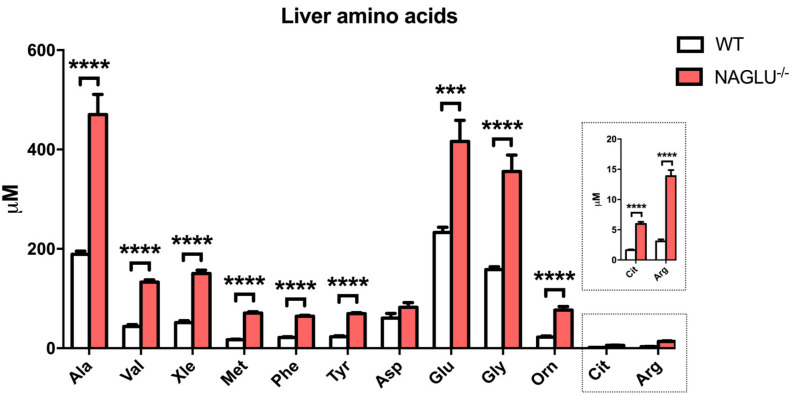
Amino acid accumulation in the liver of NAGLU^−/−^ mice. Results are represented as the mean ± standard error of the mean (SEM). The statistical significance of the difference in metabolite tissue concentrations between genotype conditions (NAGLU^−/−^ and WT) was evaluated by parametric or non-parametric tests when data failed the Shapiro–Wilk normality test. *** *p* < 0.001, **** *p* < 0.0001.

The analysis of 12 amino acids revealed significant differences in amino acid profiles in the heart and liver of NAGLU^−/−^ mice when compared to WT, although to a lesser extent in the heart (Figure 4 and Figure 5). Cardiac tissues displayed significant differences in the content of the branched-chain amino acids (BCAAs) Val, Xle, and the aromatic amino acids Phe and Tyr (Figure 4 and Table 1).

Liver tissue amino acid abundance resulted in a broad increase in NAGLU^−/−^ mice versus WT (Figure 5 and Table 2). Consistent with the heart, significant differences were observed in the levels of Val, Xle, Phe, and Tyr. In addition, all the other analyzed amino acids, except Asp, resulted in being accumulated in the liver of MPS IIIB mice.

**Figure 6:**
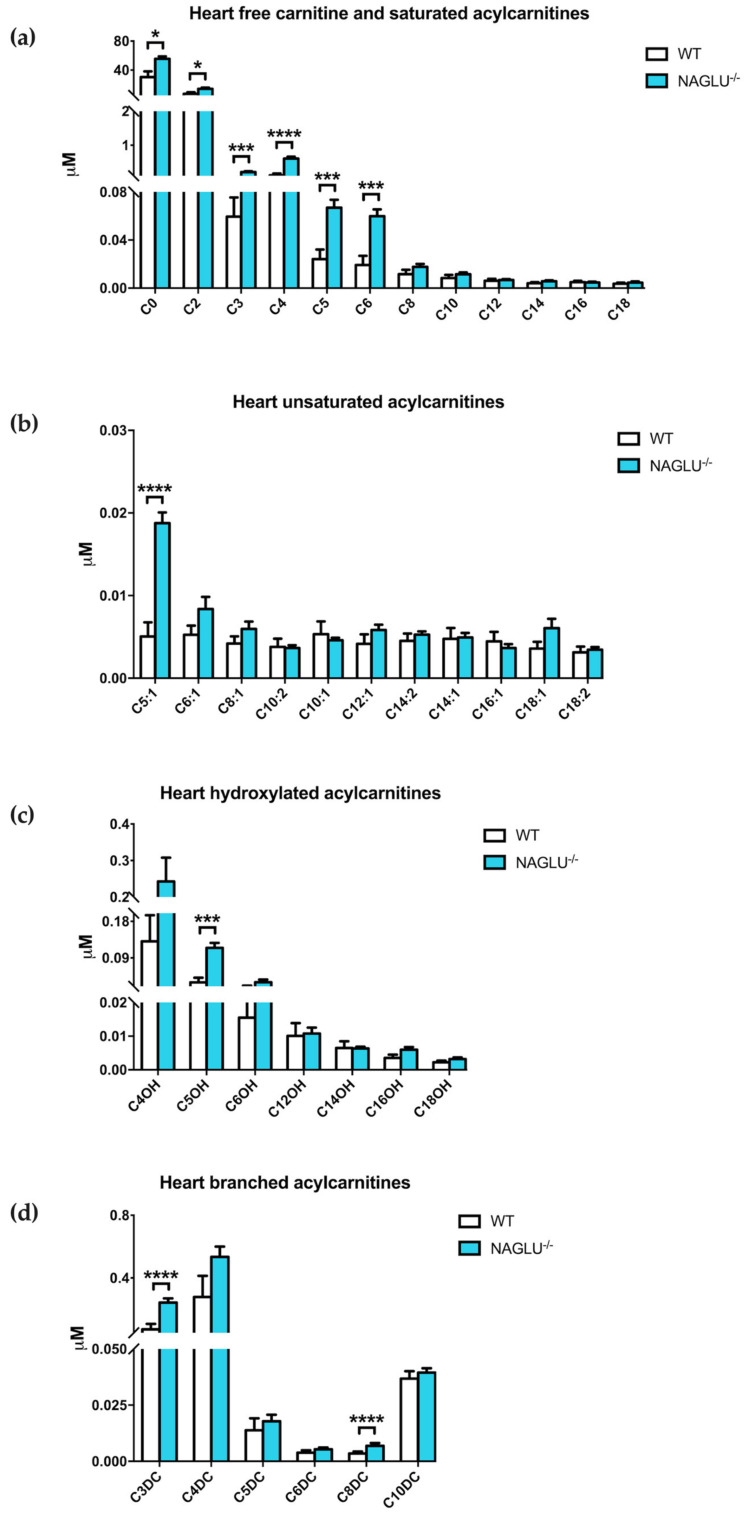
Heart-specific accumulated acylcarnitines in NAGLU^−/−^ mice: free carnitine and saturated acylcarnitine (**a**), unsaturated acylcarnitine (**b**), hydroxylated acylcarnitine (**c**) and branched acylcarnitine (**d**). Results are represented as the mean ± standard error of the mean (SEM). The statistical significance of the difference in metabolite tissue concentrations between genotype conditions (NAGLU^−/−^ and WT) was evaluated by parametric or non-parametric tests when data failed the Shapiro–Wilk normality test. * *p* < 0.05, *** *p* < 0.001, **** *p* < 0.0001.

The heart metabolome showed changes in the acylcarnitine profile in NAGLU^−/−^ mice compared to WT (Figure 6 and Table 3). Significant differences were observed in: (i) C0, free carnitine; (ii) saturated acylcarnitines: C2, acetylcarnitine; C3, propionylcarnitine; C4, butyrylcarnitine; C5, isovalerylcarnitine; C6, hexanoylcarnitine; (iii) unsaturated acylcarnitines: C5:1, 3-methylcrotonylcarnitine; (iv) hydroxylated acylcarnitines: C5OH, 3-hydroxy-isovalerylcarnitine; and (v) branched acylcarnitines: C3DC, malonylcarnitine; C8DC, suberylcarnitine.

**Figure 7:**
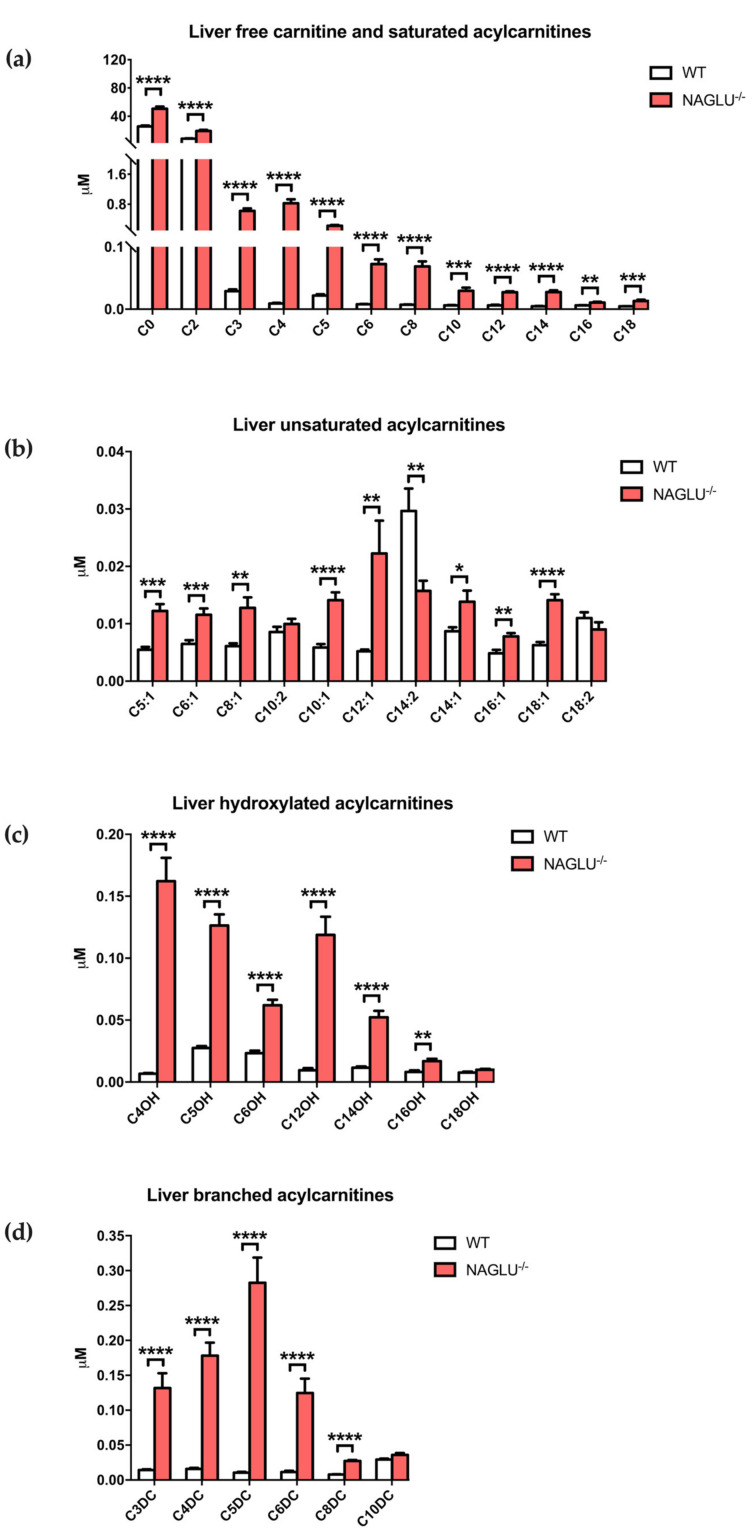
Liver-specific accumulated acylcarnitines in NAGLU^−/−^ mice: free carnitine and saturated acylcarnitine (**a**), unsaturated acylcarnitine (**b**), hydroxylated acylcarnitine (**c**) and branched acylcarnitine (**d**). Results are represented as the mean ± standard error of the mean (SEM). The statistical significance of the difference in metabolite tissue concentrations between genotype conditions (NAGLU^−/−^ and WT) was evaluated by parametric or non-parametric tests when data failed the Shapiro–Wilk normality test. * *p* < 0.05, ** *p* < 0.01, *** *p* < 0.001, **** *p* < 0.0001.

The liver metabolome showed changes in the acylcarnitine profile in NAGLU^−/−^ mice compared to WT (Figure 7 and Table 4). Significant differences were observed in: (i) C0, free carnitine; (ii) saturated acylcarnitines: C2, acetylcarnitine; C3, propionylcarnitine; C4, butyrylcarnitine; C5, isovalerylcarnitine; C6, hexanoylcarnitine; C8, octanoylcarnitine; C10, decanoylcarnitine; C12, dodecanoylcarnitine; C14, tetradecanoylcarnitine; C16, palmitoylcarnitine; C18, stearoylcarnitine; (iii) unsaturated acylcarnitines: C5:1, 3-methylcrotonylcarnitine; C6:1, 2-hexenoylcarnitine; C8:1, octenoylcarnitine; C10:1, decenoylcarnitine; C12:1, dodecenoylcarnitine; C14:1, tetradecenoylcarnitine; C14:2, tetradecadienoylcarnitine; C16:1, hexadecenoylcarnitine; C18:1, octadecenoylcarnitine; (iv) hydroxylated acylcarnitines: C4OH, 3-hydroxy-butyrylcarnitine; C5OH, 3-hydroxy-isovalerylcarnitine; C6OH, 3-hydroxy-hexanoylcarnitine; C12OH, 3-hydroxy-dodecanoylcarnitine; C14OH, 3-hydroxy-tetradecanoylcarnitine; C16OH, 3-hydroxy-palmitoylcarnitine; and (v) branched acylcarnitines: C3DC, malonylcarnitine; C4DC, methylmalonylcarnitine; C5DC, glutarylcarnitine; C6DC, methylglutarylcarnitine; C8DC, suberylcarnitine.

### 2.3. Pathway Analysis and Discriminant Metabolites Identification

**Figure 8:**
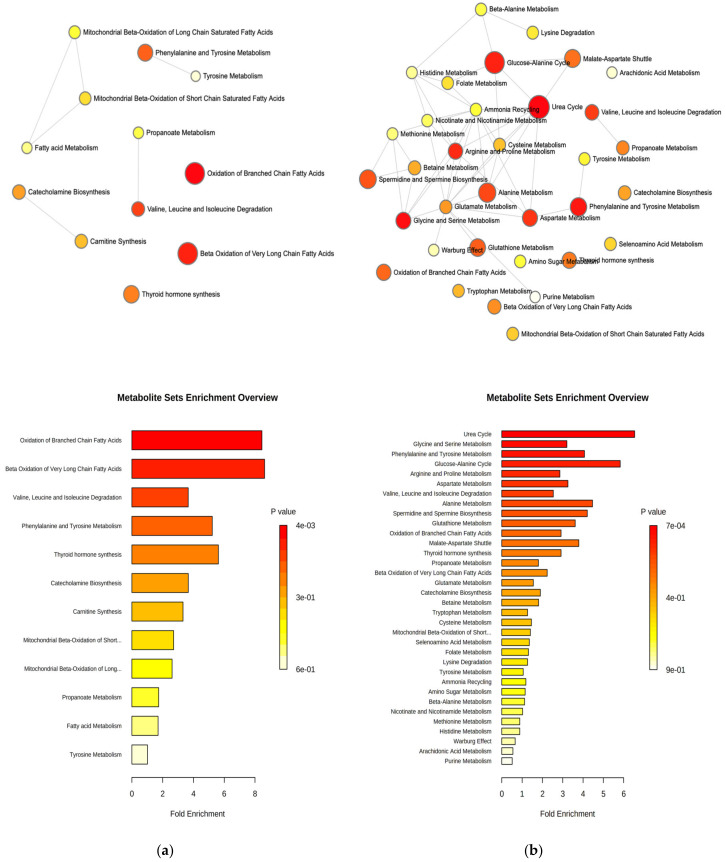
Metabolite set enrichment analysis based on the differentially abundant metabolites identified in the heart (**a**) and liver (**b**) of NAGLU^−/−^ mice.

In order to identify the principal impaired metabolic processes affected by the absence of NAGLU in the heart and liver, the quantitative data obtained by the univariate analysis were used to explore dysregulated metabolic pathways. The comparative analysis of NAGLU^−/−^ versus the control revealed the deregulation of three main metabolic pathways in the heart: the “oxidation of branched-chain fatty acids”, “beta oxidation of very-long-chain fatty acids”, and “valine, leucine and isoleucine degradation” pathways (Figure 8a). In the liver, the absence of NAGLU seems to cause dysregulation of the following pathways: “urea cycle”, “glycine and serine metabolism”, “phenylalanine and tyrosine metabolism”, “glucose-alanine cycle,” “arginine and proline metabolism”, “aspartate metabolism”, “valine, leucine and isoleucine degradation” and “alanine metabolism” (Figure 8b).

**Figure 9:**
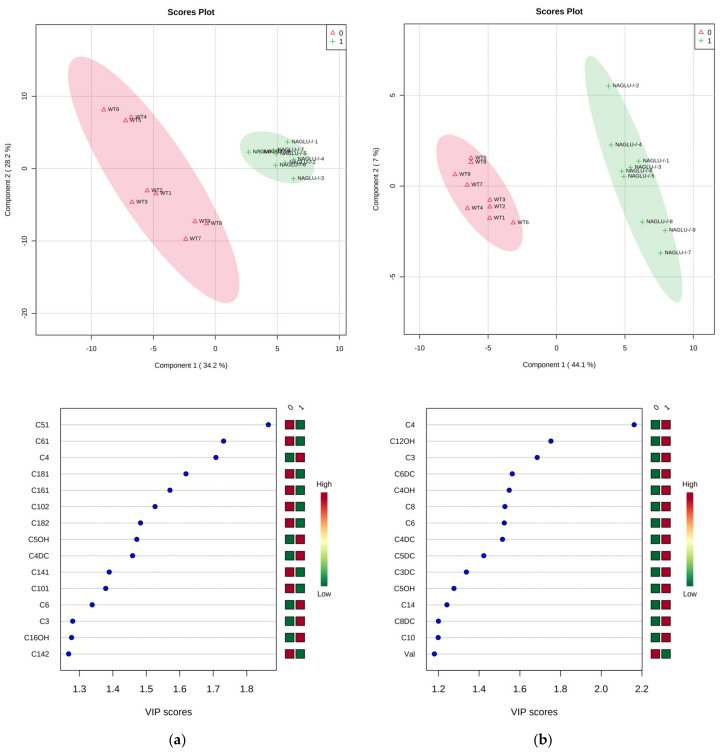
Partial least squares-discriminant analysis (PLS-DA) score plot of all metabolite features (top), and essential features identified by PLS-DA (bottom) according to the variable importance on projection (VIP) score in the heart (**a**) and liver (**b**) of the NAGLU^−/−^ differential metabolome. The concentrations of the metabolites from NAGLU^−/−^ and WT heart and liver were normalized according to protein tissue contents, log(2) transformed, and Pareto scaled. The top 15 important metabolites are summarized on the left according to their VIP score value. The intensity of the colored boxes indicates the relative metabolite abundance in each group (0 WT and 1 NAGLU^−/−^).

Finally, in order to identify the metabolites most closely associated with NAGLU deficiency, a supervised classification through a partial least squares-discriminant analysis (PLS-DA) was performed (Figure 9a,b, upper panels). The supervised analysis allowed us to define the most discriminant metabolites between NAGLU^−/−^ versus WT in heart and liver tissue, extracting a combination of variables that can be considered to have predictive value. Among them, the critical metabolites in heart and liver tissue were then estimated according to their Variable Importance in Projection (VIP) score. From the PLS-DA, the differentiation between NAGLU^−/−^ and WT genotypes occurred mainly along component 1, measured as 34.2% and 44.1% in the heart and liver tissues, respectively. The most important features identified by the PLS-DA and the VIP score plot (threshold > 1.5) included six metabolites in the heart, C5:1, C6:1, C4, C18:1, C16:1, and C10:2, and eight key metabolites in the liver, C4, C12OH, C3, C6DC, C4OH, C8, C6, and C4DC, as potentially closely associated with NAGLU deficiency (Figure 9a,b, lower panels).

## 3. Discussion

This study represents the first metabolomics-based investigation of the heart and liver of MPS IIIB-affected mice. The results obtained demonstrate that lysosomal HS storage triggers a metabolic imbalance in these organs, which is likely to contribute to disease progression. Given the resemblance of the MPS IIIB mouse model to the human disease, we anticipate that the metabolic impairment observed in the liver and heart of mice may also occur in patients with MPS IIIB, thus accounting for clinical manifestations affecting these organs.

The present study shows that NAGLU^−/−^ mice had increased levels of BCAAs (Leu, Ile, and Val), Phe and Tyr amino acids, free carnitine, and acylcarnitines in both liver and heart tissue. The augmented levels of BCAAs concomitant with the accumulation of short-chain acylcarnitines (C3, C5), involved in BCAA metabolism [33,34], suggest an impaired catabolism of BCAAs in the liver and heart of MPS IIIB-affected mice. BCAA metabolism occurs in the liver; however, it is also active in other tissues, including the heart, adipose tissues, kidney, and skeletal muscle [35]. Furthermore, the increased level of most of the acylcarnitines in the liver also suggests a deregulation of the β-oxidation of long-chain fatty acids in MPS IIIB mice, in agreement with the primary function of acylcarnitines of serving as carriers to transport long-chain fatty acids to mitochondria for subsequent β-oxidation, thus providing energy for cell activities.

In the liver tissue of MPS IIIB mice, as well as the amino acids discussed above, we also found elevated levels of the Ala, Met, Glu, Gly, Arg, Orn, and Cit amino acids. These results suggest a deregulation of the glucose-alanine cycle and the urea cycle in the liver of MPS IIIB mice, as well as the altered BCAA and other amino acid metabolisms. These results are consistent with a previous metabolite analysis of the liver of MPS I mice showing decreased levels of lipids, simple carbohydrates, and nucleotides, whereas most amino acids, amino acid derivatives, dipeptides, and urea resulted in being increased, suggesting an increase in protein catabolism [36]. Although some results have been reported in previous global metabolomic profiling of MPS IIIB mice and patients, where a reduction of all key amino acids was observed in the serum of affected subjects as compared to controls, probably due to either differences in the methodological strategies or metabolic differences in the tested tissues [29,37], our findings are definitely in agreement with previous metabolic phenotyping performed on the urine of MPS III (A–D) and MPS VI patients [31,32].

The enhancement of protein catabolism in MPS IIIB mouse tissue at least in part may fulfill the intermediary metabolism in response to carbohydrate and lipid deficiencies. Indeed, inside the cells, BCAAs can be stored in amino acid pools, integrated into proteins, or shuttled to mitochondria for oxidation. They serve as signaling molecules regulating glucose and lipid metabolism as well as protein synthesis through the phosphoinositide 3-kinase/protein kinase B/mammalian target of the rapamycin (PI3K/AKT/mTOR) signal pathway [38,39]. In particular, the final metabolites produced in the catabolism of BCAAs provide an energy supply. The terminal metabolites of the catabolism of Leu are acetoacetate and acetyl-CoenzymeA (CoA), which are ketogenic; Ile yields to propionyl-CoA and acetyl-CoA, which are both glucogenic and ketogenic, and Val produces succinyl-CoA, which is glucogenic. Acetyl-CoA can enter the tricarboxylic acid cycle (TCA), while succinyl-CoA and acetoacetate are TCA intermediates. These findings are reinforced by previous studies on correlated disease models. In MPS I, MPS IIIB, MPS VII, Niemann–Pick type A/B, and infantile neuronal ceroid lipofuscinosis mouse models, a significantly reduced adiposity was detected, suggesting that the reduction of lysosomal stored material recycling results in an energy imbalance with an overall impact on autophagy levels and metabolism [36,40,41].

Multiple reports show that an impairment of autophagy by engulfed lysosomes drives the progression of the disease in LSDs [3,4,5,42,43,44,45]. Autophagy is a cellular process that targets cytoplasmic material such as long-lived proteins and organelles to lysosomes for degradation into simpler metabolites [46]. Autophagy activation is regulated by the mechanistic target of rapamycin complex 1 (mTORC1), whose activity is dependent on a variety of inputs from energy, growth factors, oxygen, and nutrients [47,48]. Upon activation, mTORC1 promotes anabolic processes while blocking autophagy and other catabolic processes. Experimental studies, showing that amino acid withdrawal from cells suppresses mTORC1 signaling and triggers autophagy, have highlighted the regulatory role of cellular amino acids in mTORC1 activity [49]. These findings are consistent with the identification of a group of small GTPases, Rags, as central mediators of amino acid signaling for mTORC1 [50,51]. Recently, a definite role has emerged for the amino acid/mTORC1 pathway centered at the lysosomes in the control of autophagy [52].

Amino acids promote the localization of mTORC1 at the lysosomes and induce its activation through both Rag-dependent and Rag-independent pathways [51,53,54]. Lysosomal localization and the subsequent activation of mTORC1 involves two concerted events: sensitization by priming amino acids and subsequent activation by activating amino acids [55]. The BCAAs Leu, Ile and Val belong to the group of activating amino acids, and Leu is among the main positive regulator of mTORC1 signaling. When activated, mTORC1 stimulates protein, lipid, and nucleotide biosynthesis by phosphorylating several downstream targets, while inhibiting cellular catabolism through the repression of autophagy [56]. Hyperactivation of mTORC1 associated with the suppression of autophagy has been recognized as the main pathological mechanism through which lysosomal storage impairs chondrocyte function and bone growth in MPS VII [45]. On the other hand, defective autophagic flux has been demonstrated in the brain of mouse models of multiple sulfatase deficiency (MSD), MPS IIIA, and Nieman-Pick type C (NPC) disease, and in human fibroblasts from patients affected by MPS VI, Gaucher disease, Fabry disease, and other LSDs [57]. Inhibitors of mTORC1, which stimulate autophagy, have been successfully tested in models of some LSDs as a potential therapeutic intervention [4,5]. Thus, the imbalance of BCAAs and other amino acid levels in the NAGLU^−/−^ mouse heart correlates well with our previous findings demonstrating an impaired lysosomal autophagic flux associated with cardiac phenotype in MPS IIIB mice [9].

Notably, lysosomal mTORC1 and Rag GTPases have been shown to regulate the localization of the transcription factor EB (TFEB) involved in the lysosomal biogenesis and autophagy, by phosphorylating it on several serine and threonine residues [58]. When phosphorylated by mTORC1, TFEB is retained in the cytoplasm. On the contrary, mTORC1 inactivation induces the dephosphorylation of TFEB with consequent relocalization to the nucleus, where TFEB promotes the expression of lysosomal and autophagic-related genes, thus contributing to the promotion of autophagic flux, the biogenesis of new lysosomes, and the clearance of storage material [59]. The amino acid pathway is the main orchestrator in regulating the activity of TFEB, and its impaired signaling has been shown to highly contribute to the pathogenesis of LSDs [60]. Thus, we can speculate that the altered levels of BCAAs, other amino acids, and acylcarnitines in the heart and liver of MPS IIIB mice are likely to deregulate mTORC1 and its downstream target TFEB, thus perturbing the autophagic process in these tissues.

An impairment of autophagy is usually associated with the dysregulation of mitochondrial quality control pathways and the accumulation of damaged mitochondria within the cells. In LSDs, reduced autophagic flux has been shown to lead to the persistence of dysfunctional mitochondria with a consequent increase in reactive oxygen species production, altered calcium homeostasis, and enhanced pro-apoptotic signals [43,61,62]. Moreover, many studies have shown that defective mitochondrial activity contributes to neuroinflammation and cognitive defects in different MPS III mouse models [63,64]. These findings on the diffuse presence of defective mitochondria in many MPS diseases support our results relating to impaired β-oxidation-related pathways in MPS IIIB mice.

In conclusion, the metabolic phenotype observed in the liver and heart of MPS IIIB mice supports the view that autophagy and mitophagy deregulation may play a prominent role in the pathogenesis of the MPS IIIB disease. This may be relevant for the development of effective therapies for curing MPS IIIB.

## 4. Materials and Methods

### 4.1. Animals

The animal model of MPS IIIB (NAGLU knockout mice, NAGLU^−/−^) was created by Prof. Elizabeth Neufeld, UCLA, by interrupting exon 6 of the *NAGLU* gene on the C57/BL6 background [21]. NAGLU^−/−^ and WT mice were genotyped by PCR [9]. Mice (4 per cage) were maintained on a 12 h light/dark cycle, identical temperature conditions (21 ± 1 °C) and humidity (60 ± 5%), and free access to normal mouse chow [65,66]. The experimental protocols were carried out following ARRIVE guidelines and EU Directive 2010/63/EU for animal experiments. All mouse care and handling procedures were approved by the Institutional Animal Care and Use Committee (IACUC) of the Biotechnology Center, AORN Cardarelli (Naples, Italy), project identification code C6AC4.4 (19 February 2016). The sacrifice of the animals was performed in the morning to avoid variation in sample collection due to time [67,68]. Firstly, 8-month-old animals were euthanized, and then the thoracic and abdominal cavities were opened to expose the heart and liver. Organs were perfused by PBS in order to eliminate blood residue in the vessels of the collected tissues. The organs, liver and heart, were rapidly removed, washed with ice-cold PBS, and stored at −80 °C.

### 4.2. Extraction and Derivatization of the Metabolites

Metabolite extraction from the heart and liver tissue of nine MPS IIIB and nine WT male mice for liquid chromatography–tandem mass spectrometry (LC-MS/MS) analysis was performed according to published methods, with little modification [69,70]. The frozen tissues were homogenized in 1000 μL of 50:50 cold methanol/0.1 M hydrogen chloride (HCl) (Sigma-Aldrich, St. Louis, MO, USA). Mechanical homogenization was performed using a TissueLyser LT homogenizer (Qiagen, Duesseldorf, Germany) at high speed, shaking in 2 mL microcentrifuge tubes and with stainless-steel beads. The mixture was centrifuged at 13,000 rpm for 60 min at 4 °C to isolate metabolites from the protein pellet. Proteins were extracted from the pellet to estimate protein concentration, as explained [71]. The supernatant, containing metabolites, was treated with potassium hydroxide (Sigma-Aldrich, St. Louis, MO, USA) until it reached pH 7–8, then centrifuged at 13,000 rpm for 40 min, cleaned by the pellet, and dried under nitrogen. The dried metabolite mixture was resuspended in 1.0 mL of methanol and analyzed by tandem mass spectrometry to determine the contents of amino acids (AAs) and acylcarnitines (ACs). The metabolomic platform was developed to perform the fast identification of biomarkers of inherited metabolomic diseases and was adapted for the purpose of the present analytical work. The chosen analytical method (neutral loss scan and precursor ion scan) is ideal for the simultaneous, fast, and quantitative measurement of a family of metabolites, such as amino acids or acylcarnitines [68,71,72].

A total of 100 µL of the standard mixture containing labeled AAs and ACs were added to 100 µL of the sample. The standard concentrations were in the 500–2500 μmol/L range for AAs and in the 7.6–152 μmol/L range for ACs. The labeled AA and AC mixtures were composed of ^15^N, 2-^13^C-Gly, ^2^H_4_-Ala, ^2^H_8_-Val, ^2^H_3_-Leu, ^2^H_3_-Met, ^13^C_6_-Phe, ^13^C_6_-Tyr, ^2^H_3_-Asp, ^2^H_3_-Glu, ^2^H_2_-Orn, ^2^H_2_-Cit (Cambridge Isotope Labs, USA), and ^2^H_4_,5-^13^C-Arg and ^2^H_9_-C0, ^2^H_3_-C2, ^2^H_3_-C3, ^2^H_3_-C4, ^2^H_9_-C5, ^2^H_3_-C8, ^2^H_9_-C14, and ^2^H_3_-C16 (Cambridge Isotope Labs, USA), respectively. The samples were shaken for 20 min at room temperature on an orbital shaking system and dried under a nitrogen flow at 40 °C. AAs and ACs were derivatized to butyl esters with 80 μL of butanol in 3N HCl (Sigma-Aldrich, St. Louis, MO, USA) at 65 °C for 25 min. The samples were dried again after derivatization and were, finally, resuspended in 300 μL of acetonitrile/water (70:30) containing 0.1% formic acid. For the MS/MS experiments, 60 μL of the final solution was injected in flow injection analysis mode. Each sample was analyzed in triplicate.

### 4.3. Metabolite LC-MS/MS Measurements

A group of metabolites constituted of 12 AAs and 35 ACs was measured employing a targeted MS-based metabolomic platform [73]. In detail, tandem mass spectrometric analysis was carried out with an API 4000 triple quadrupole mass spectrometer (Applied Biosystems-Sciex, Toronto, Canada) coupled with an 1100 series Agilent high-performance liquid chromatography system (Agilent Technologies, Waldbronn, Germany).

MS/MS analysis for Ala, Val, Xle, Met, Phe, Tyr, Asp, and Glu was conducted using a neutral loss of 102 Da scan function, according to the following parameters: polarity: positive; m/z range (Da): 130–280; declustering potential (DP) (volts): 45; collision energy (CE) (volts): 25. MS/MS analysis for Gly, Orn, Cyt, and Arg was performed in multiple reaction monitoring (MRM) mode, according to the following parameters: polarity: positive; Q1/Q3 (m/z): 132.1/76.0 (Gly), 189.1/70.0 (Orn), 231.2/70.0 (Arg), 232.2/113.1 (Cit); DP (volts): 43 (Gly), 33 (Orn), 60 (Arg), 50 (Cit); CE (volts): 14 (Gly), 33 (Orn), 45 (Arg), 28 (Cit).

Finally, a precursor scan of 85.1 Da was used for the ACs analysis, according to the following parameters: polarity: positive; m/z range (Da): 200–560; DP range (volts): 55–80; CE range (volts): 34–60.

Quantitative analysis of the data was performed with ChemoView v1.2 software through the comparison of the analyte and its corresponding internal standard areas. Using stable isotope-labeled internal standards improves correction for the matrix effect. The MS method’s accuracy and precision were estimated by analyzing quality control (QC) samples provided by ERNDIM (European research network for evaluation and improvement of screening, diagnosis, and treatment of inherited disorders of metabolism, Manchester, UK; www.erndimqa.nl), prepared at low, mid, high, and very high concentrations.

### 4.4. Metabolite Statistical Analysis and Feature Selection

The NAGLU^−/−^ and WT mouse tissue metabolite measurements were processed by univariate and multivariate analysis. Metabolite concentrations, expressed as µM, were normalized to the protein content of the analyzed tissues and referred to as 1 mg of total protein content [74].

Univariate statistics were calculated with GraphPad Prism 8.0, and the results are presented as the mean ± standard error of the mean (SEM). The statistical significance of the difference in metabolite tissue concentrations between genotype conditions (NAGLU^−/−^ and WT) was evaluated by parametric (unpaired t-test) or non-parametric (Mann–Whitney test) tests when data failed the Shapiro–Wilk normality test. Two outlier samples were removed from the metabolome analysis.

Multivariate statistical analysis and metabolite set enrichment analysis were performed using MetaboAnalyst 4.0 (http://www.metaboanalyst.ca) [75]. The normalized metabolic dataset was log(2)-transformed and scaled according to the Pareto scaling method. Dataset homogeneity was evaluated by principal component analysis (PCA) to get an overview of the data and identify potential severe outliers. Partial least squares-discriminant analysis (PLS-DA) was used as a supervised method to maximize the covariance between the independent variables (metabolites) and the corresponding dependent variable Y for predictive feature identification. The variable importance on projection (VIP) was estimated. VIP is a weighted sum of squares of the PLS loadings taking into account the amount of explained variation in each dimension. Finally, the interpretation of the metabolomic dataset was carried out using metabolite set enrichment analysis by MetaboAnalyst 4.0 (http://www.metaboanalyst.ca). The Human Metabolome Database was used to assign the correctly numbered ID (HMDB ID) to each differentially abundant metabolite. 

## 5. Conclusions

In summary, targeted metabolomic profiling demonstrates elevated levels of BCAAs, other amino acids, and acylcarnitines in the liver and heart of the MPS IIIB mouse model. We also identified impaired metabolic pathways such as BCAA catabolism and phenylalanine and tyrosine metabolism in both the liver and heart; branched- and long-chain fatty acid β-oxidation in the heart; and mainly the urea cycle and the glucose-alanine cycle in the liver of MPS IIIB mice. We speculate that the secondary accumulation of BCAAs and other amino acids in the liver and the heart of MPS IIIB mice affects autophagy, which in turn leads to altered cellular metabolism, thus contributing to the progression of the disease.

## Figures and Tables

**Table 1:** Differentially abundant metabolites in the heart.

	Amino Acid Concentrations		*p-*Value
	WT	NAGLU^−/−^	
Val	24.4 ± 5.2	38.3 ± 3.2	0.0278
Xle	33.4 ± 8.1	57.4 ± 5.2	0.0164
Phe	9.8 ± 2.2	20.2 ± 1.6	0.0008
Tyr	8.9 ± 2.6	17.2 ± 1.5	0.0059

Concentrations are expressed as µM. Abbreviations: Val, Valine; Xle, Leucine and/or Isoleucine; Phe, Phenylalanine; Tyr, Tyrosine.

**Table 2:** Differentially abundant metabolites in the liver.

	Amino Acid Concentrations		*p-*Value
	WT	NAGLU^−/−^	
Ala	189.1± 6.2	470.4 ± 40.4	<0.0001
Val	43.9 ± 3.8	133.1 ± 4.5	<0.0001
Xle	51.5 ± 4.0	150.3 ± 6.8	<0.0001
Met	17.0 ± 1.3	70.6 ± 3.0	<0.0001
Phe	21.5 ± 1.5	64.4 ± 2.0	<0.0001
Tyr	23.0 ± 1.9	69.8 ± 1.8	0.0007
Glu	232.8 ± 10.8	416.2 ± 42.4	<0.0001
Gly	158.1 ± 5.6	355.6 ± 33.2	<0.0001
Orn	22.4 ± 2.0	76.8 ± 7.1	<0.0001
Cit	1.6 ± 0.1	6.0 ± 0.3	<0.0001
Arg	3.1 ± 0.3	13.9 ± 1.0	<0.0001

Concentrations are expressed as µM. Abbreviations: Ala, Alanine; Val, Valine; Xle, Leucine and/or Isoleucine; Met, Methionine; Phe, Phenylalanine; Tyr, Tyrosine; Glu, Glutamic acid; Gly, Glycine; Orn, Ornitine, Cit, Citrulline, Arg, Arginine.

**Table 3:** Differentially abundant metabolites in the heart.

	Saturated Acylcarnitine Concentrations		*p-*Value
	WT	NAGLU^−/−^	
C0	31.1808 ± 7.8309	55.4627 ± 3.5891	0.0103
C2	8.1067 ± 1.9993	14.0801 ± 1.7085	0.0205
C3	0.0763 ± 0.0160	0.2101 ± 0.0256	0.0001
C4	0.1532 ± 0.0539	0.6067 ± 0.0557	<0.0001
C5	0.0258 ± 0.0079	0.0669 ± 0.0065	0.0007
C6	0.0235 ± 0.0075	0.0599 ± 0.0057	0.0005
	**Unsaturated Acylcarnitine Concentrations**		
C5:1	0.0063 ± 0.0017	0.0188 ± 0.0013	<0.0001
	**Hydroxylated Acylcarnitine Concentrations**		
C5OH	0.0395 ± 0.0115	0.1147 ± 0.0125	0.0001
	**Branched Acylcarnitine Concentrations**		
C3DC	0.0926 ± 0.0348	0.2430 ± 0.0268	<0.0001
C8DC	0.0035 ± 0.0008	0.0069 ± 0.0013	<0.0001

Concentrations are expressed as µM. Abbreviations: C0, free carnitine; C2, acetylcarnitine; C3, propionylcarnitine; C4, butyrylcarnitine; C5, isovalerylcarnitine; C6, hexanoylcarnitine; C5:1, 3-methylcrotonylcarnitine; C5OH, 3-hydroxy-isovalerylcarnitine; C3DC, malonylcarnitine; C8DC, suberylcarnitine.

**Table 4:** Differentially abundant metabolites in the liver.

	Saturated Acylcarnitine Concentrations		*p-*Value
	WT	NAGLU^−/−^	
C0	25.5149 ± 1.4088	50.6110 ± 3.1383	<0.0001
C2	8.3791 ± 0.3831	19.1520 ± 1.4497	<0.0001
C3	0.0290 ± 0.0028	0.6242 ± 0.0672	<0.0001
C4	0.0092 ± 0.0008	0.8251 ± 0.1065	<0.0001
C5	0.0219 ± 0.0019	0.2256 ± 0.0234	<0.0001
C6	0.0078 ± 0.0006	0.0722 ± 0.0075	<0.0001
C8	0.0071 ± 0.0004	0.0686 ± 0.0079	<0.0001
C10	0.0060 ± 0.0005	0.0294 ± 0.0050	0.0003
C12	0.0061 ± 0.0011	0.0271 ± 0.0016	<0.0001
C14	0.0047 ± 0.0005	0.0274 ± 0.0027	<0.0001
C16	0.0061 ± 0.0004	0.0107 ± 0.0013	0.0028
C18	0.0047 ± 0.0003	0.0132 ± 0.0021	0.0008
	**Unsaturated Acylcarnitine Concentrations**		
C5:1	0.0055 ± 0.0005	0.0122 ± 0.0012	0.0001
C6:1	0.0065 ± 0.0007	0.0116 ± 0.0011	0.001
C8:1	0.0061 ± 0.0005	0.0128 ± 0.0018	0.0028
C10:1	0.0058 ± 0.0006	0.0141 ± 0.0014	<0.0001
C12:1	0.0052 ± 0.0003	0.0222 ± 0.0057	0.0091
C14:2	0.0297 ± 0.0039	0.0157 ± 0.0018	0.0051
C14:1	0.0087 ± 0.0007	0.0139 ± 0.0019	0.0234
C16:1	0.0049 ± 0.0006	0.0078 ± 0.0006	0.0027
C18:1	0.0063 ± 0.0005	0.0141 ± 0.0010	<0.0001
	**Hydroxylated Acylcarnitine Concentrations**		
C4OH	0.0068 ± 0.0005	0.1621 ± 0.0189	<0.0001
C5OH	0.0275 ± 0.0015	0.1263 ± 0.0090	<0.0001
C6OH	0.0234 ± 0.0019	0.0621 ± 0.0043	<0.0001
C12OH	0.0096 ± 0.0017	0.1188 ± 0.0146	<0.0001
C14OH	0.0115 ± 0.0011	0.0522 ± 0.0052	<0.0001
C16OH	0.0082 ± 0.0012	0.0168 ± 0.0020	0.0018
	**Branched Acylcarnitine Concentrations**		
C3DC	0.0143 ± 0.0011	0.1317 ± 0.0214	<0.0001
C4DC	0.0158 ± 0.0015	0.1781 ± 0.0186	<0.0001
C5DC	0.0104 ± 0.0012	0.2825 ± 0.0363	<0.0001
C6DC	0.0113 ± 0.0018	0.1246 ± 0.0206	<0.0001
C8DC	0.0078 ± 0.0005	0.0273 ± 0.0014	<0.0001

Concentrations are expressed as µM. Abbreviations: C8, octanoylcarnitine; C10, decanoylcarnitine; C12, dodecanoylcarnitine; C14, tetradecanoylcarnitine; C16, palmitoylcarnitine; C18, stearoylcarnitine; C6:1, 2-hexenoylcarnitine; C8:1, octenoylcarnitine; C10:1, decenoylcarnitine; C12:1, dodecenoylcarnitine; C14:1, tetradecenoylcarnitine; C14:2, tetradecadienoylcarnitine; C16:1, hexadecenoylcarnitine; C18:1, octadecenoylcarnitine; C4OH, 3-hydroxy-butyrylcarnitine; C6OH, 3-hydroxy-hexanoylcarnitine; C12OH, 3-hydroxy-dodecanoylcarnitine; C14OH, 3-hydroxy-tetradecanoylcarnitine; C16OH, 3-hydroxy-palmitoylcarnitine; C4DC, methylmalonylcarnitine; C5DC, glutarylcarnitine; C6DC, methylglutarylcarnitine.

## Supplementary Materials

Supplementary Materials can be found at https://www.mdpi.com/1422-0067/21/12/4211/s1. Table S1: Heart metabolite concentrations (μM); Table S2: Liver metabolite concentrations (μM).
